# Organic vs. Conventional Grassland Management: Do ^15^N and ^13^C Isotopic Signatures of Hay and Soil Samples Differ?

**DOI:** 10.1371/journal.pone.0078134

**Published:** 2013-10-25

**Authors:** Valentin H. Klaus, Norbert Hölzel, Daniel Prati, Barbara Schmitt, Ingo Schöning, Marion Schrumpf, Markus Fischer, Till Kleinebecker

**Affiliations:** 1 University of Münster, Institute of Landscape Ecology, Münster, Germany; 2 University of Bern, Institute of Plant Sciences, Bern, Switzerland; 3 Max-Planck-Institute for Biogeochemistry, Jena, Germany; North Carolina State University, United States of America

## Abstract

Distinguishing organic and conventional products is a major issue of food security and authenticity. Previous studies successfully used stable isotopes to separate organic and conventional products, but up to now, this approach was not tested for organic grassland hay and soil. Moreover, isotopic abundances could be a powerful tool to elucidate differences in ecosystem functioning and driving mechanisms of element cycling in organic and conventional management systems. Here, we studied the δ^15^N and δ^13^C isotopic composition of soil and hay samples of 21 organic and 34 conventional grasslands in two German regions. We also used Δδ^15^N (δ^15^N plant - δ^15^N soil) to characterize nitrogen dynamics. In order to detect temporal trends, isotopic abundances in organic grasslands were related to the time since certification. Furthermore, discriminant analysis was used to test whether the respective management type can be deduced from observed isotopic abundances.

Isotopic analyses revealed no significant differences in δ^13^C in hay and δ^15^N in both soil and hay between management types, but showed that δ^13^C abundances were significantly lower in soil of organic compared to conventional grasslands. Δδ^15^N values implied that management types did not substantially differ in nitrogen cycling. Only δ^13^C in soil and hay showed significant negative relationships with the time since certification. Thus, our result suggest that organic grasslands suffered less from drought stress compared to conventional grasslands most likely due to a benefit of higher plant species richness, as previously shown by manipulative biodiversity experiments. Finally, it was possible to correctly classify about two third of the samples according to their management using isotopic abundances in soil and hay. However, as more than half of the organic samples were incorrectly classified, we infer that more research is needed to improve this approach before it can be efficiently used in practice.

## Introduction

Distinguishing organic and conventional products is a major issue of food security and authenticity and much research on method development has been conducted to tackle this issue [1]. Stable isotope analysis was proven to give important insight in ecosystem functioning and was successfully used to detect differences between organic and conventional agriculture [2], [3], [4]. Since Nakano et al. [5] proposed the use of natural abundances of stable isotopes to separate organic and conventional products, several studies tested this approach successfully for fruits, vegetables and other plant products [1], [6], [7], [8], [9], [10] as well as for beef [11], [12] and milk [13], but not for grassland hay or soil samples. Differences among organic and conventional plant products were mostly attributed to differences in δ^15^N isotopic signatures of applied fertilizers [14], because organic farming abandons the use of synthetic mineral fertilizers. While such conventional (synthetic) N sources exhibit δ^15^N values close to 0‰, organic N sources such as cattle dung or slurry are strongly enriched in δ^15^N [15]. Consequently, organic farming products are mostly enriched in δ^15^N compared to conventional ones due to the replacement of synthetic N sources by organic fertilizers. However, in nature δ^15^N abundances in plants are affected by a multiplicity of factors such as type and degree of mycorrhization, the chemical type of N-compounds taken up or further soil characteristics, which can be barely separated from each other [16], [17].

Similarly, δ^13^C in plant and soil are also of broad ecological interest [18]. In C3 plants, which represent Central European grassland vegetation, δ^13^C abundances in biomass are first of all affected by water availability and drought stress, but show also significant interactions with nutrient availability and fertilization [2], [19]. Additionally, δ^13^C values are related to a different contribution of CO_2_ from soil respiration to plant photosynthesis y [6], [20] and thus contain valuable ecological information related to agricultural management. Furthermore, was shown to be related to functional aspects of plant communities [18].

Although grasslands play a central role in the production of organic meat and dairy products [21], and proportions of organic grasslands have increased significantly during the last decade [22], stable isotope analysis was so far not used to distinguish between soils and yield (hay) of organically and conventionally managed grasslands. As organic fertilizers can even in grasslands lead to higher δ^15^N values in soil and vegetation [23], this might give the ability to classify organic and conventional plant products using isotopic abundances [7]. Moreover, isotopic abundances are related to important ecosystem processes affecting nutrient cycling and balances [24] and thus bear the potential to elucidate possible differences in ecosystem functioning of organic vs. conventional grasslands, which are otherwise difficult to detect.

Here, we studied 21 organic and 34 conventional grasslands in two German regions and analyzed δ^13^C and δ^15^N of soil and hay (plant biomass). Furthermore, Δδ^15^N values (δ^15^N plant - δ^15^N soil) were calculated to estimate differences in nitrogen dynamics. We also assessed the time since organic certification to test for temporal trends. In detail, we analyzed whether (a) differences in isotopic abundances among organic and conventional grasslands exist and whether there are (b) significant trends in isotopic composition with time since conversion to organic management. Additionally (c), we used discriminant analysis to deduce the respective management type from the isotopic composition of hay and/or soil samples.

## Methods

### Ethics statement

Field work permits were given by the responsible state environmental offices of Baden-Württemberg, Thüringen, and Brandenburg (according to § 72 BbgNatSchG).

### Study design

We studied agriculturally used permanent grasslands in two regions in Germany which belong to the *Biodiversity Exploratories* project [25]: (I) *Hainich-Dün* in Thuringia in central Germany situated in and around the National Park Hainich and (II) the Biosphere Reserve *Schwäbische Alb* in Baden-Württemberg in south-west Germany. In grasslands of both regions Cambisols occur, while in the Schwäbsiche Alb Leptosols and in Hainich-Dün Stagnosols and Vertisols can also be found. Grassland types could be categorized as pastures, meadows and mown pastures [25]. To get information on land use for each grassland, farmers and land owners were annually questioned about the amount and type of fertilizer (kg N ha^−1^) from 2006 to 2010 [26]. We chose organic and conventional grasslands from a randomly selected dataset of 50 plots in each region. Organic management of grasslands abandons pesticides and synthetic fertilizers, restricts livestock density and the use of organic fertilizers from animal husbandry to a maximum of 170 kg N*ha^−1^*a^−1^ (European Union, 2008). Accordingly, study plots can be distinguished in two sub-sets: organic plots which are managed according to an official organic farming certificate [27] and uncertified (conventional) plots, where management goes against certification criteria. Please note that all unfertilized but not certified grasslands were excluded from the analysis. Finally, we used 17 conventional plots per study region as well as 17 organic plots at Hanich-Dün and 4 organic plots at the Schwäbische Alb for comparison. Duration of organic management of the grasslands differs from 3 up to 20 years. Although we have no detailed data on the management prior conversion to organic farming, it seems to be likely that at least some of the organic grasslands were already previously managed at a low to medium intensity. The proportion of legumes varied widely among study plots (from 0.0 to 60.5%) but not among organic and conventional management (data not shown). C4 plants are generally no regular component of Central European grassland vegetation.

### Field work and chemical analyses

Soil sampling was conducted in early May 2011. On each plot mixed samples of 14 soil cores from 0 to 10 cm depth were collected using a split tube sampler with a diameter of 5 cm. Cores were taken along two 20 m transects at each plot. Roots were removed from the samples in the field and soil samples were air-dried, sieved to <2 mm and ground. For δ13C analyses, soil samples were weighted into tin capsules and treated with sulphurous acid inside the capsules to remove carbonates. Samples were dried again at 70°C before combustion in an oxygen stream using an elemental analyser (NA 1110, CE Instruments, Milan, Italy). Evolved CO2 was analysed using an isotope ratio mass spectrometer (IRMS; Delta C or DELTA+XL, Thermo Finnigan MAT, Bremen, Germany). For biomass sampling, we harvested aboveground community biomass in four quadrates of 0.25 m^2^ from mid-May to mid-June 2011 in both regions simultaneously. Temporary fences ensured that no mowing or grazing took place before yield was sampled. Plant material was dried for 48 h at 80°C and ground to fine powder for lab analyses. Both isotopic abundances of biomass samples and δ^15^N of soil samples were determined by mass spectrometry (Finnigan MAT DeltaPlus with Carlo Erba Elementar Analysator with ConFlo II Interface). Isotope ratios are given in per mille (‰), whereby δ^13^C is relative to the international reference standard v-PDB using NBS19 and δ^15^N relative to AIR-N_2_ [28].

The nearby climate station in *Hainich-Dün*, located in Schönstedt (193 m A.S.L.), revealed that the precipitation prior to sampling (April and May 2011) was 70% lower than the mean of the same month from 2003–2010 (33.8 mm instead of 116.2 mm) [29]. At the *Schwäbische Alb*, the next climate station is situated in Münsingen-Rietheim (732 m A.S.L). Compared to the same month in the period 2005–2010, precipitation in April and May 2011 was 20% lower (153.8 mm instead of 191.5 mm) [30].

### Data analysis

Multiple analysis of variance (ANOVA) models were used to examine differences among management types (organic vs. conventional) while also accounting for farm or land owner (*n* = 7), soil type (*n* = 4), study region (*n* = 2) and grasslands type (meadow, pasture, mown pasture) including all two-way interactions with management type. Therefore, the lm() and step() functions in R for stepwise reduction of explanatory variables were used. Obtaining model results by using the anova() output assured that the order of variables entering the model had no effect on significance gained for the respective variable, because all other variables were taken into account previously as co-variables. Model assumptions were checked using diagnostic plots function. Similarly, also applying the lm() function analysis of co-variance (ANCOVA) models were calculated to relate the isotopic composition to the time since certification in all organic plots including farm, soil type and study region. Soil texture (proportions of clay, silt and sand) was also incorporated in lm() models but removed for final analysis as they did not add to explained variance. Additionally, we employed the qda() function from the MASS package [31] to perform Quadratic Discriminant Analysis for the classification of organic and conventional grasslands based on ^15^N and ^13^C isotopic values in soil and biomass samples. To ensure normal distribution of variables log10 transformation was applied prior to all analyses where necessary. All statistical tests were performed with R [32].

## Results

Farmer's questionnaires revealed that organic grasslands received average fertilizer applications of 21.6 (±37.0) kg N a^−1^, while conventional grasslands received more than the threefold amount: 72.3 (±41.5) kg N a^−1^. However, both management systems show wide variation in fertilization intensity at the plot level. Organic plots received only organic fertilizers, as prescribed by organic management guidelines, whereas conventional plots received mostly mineral fertilizers (75% of total fertilizer N).

**Figure 1:**
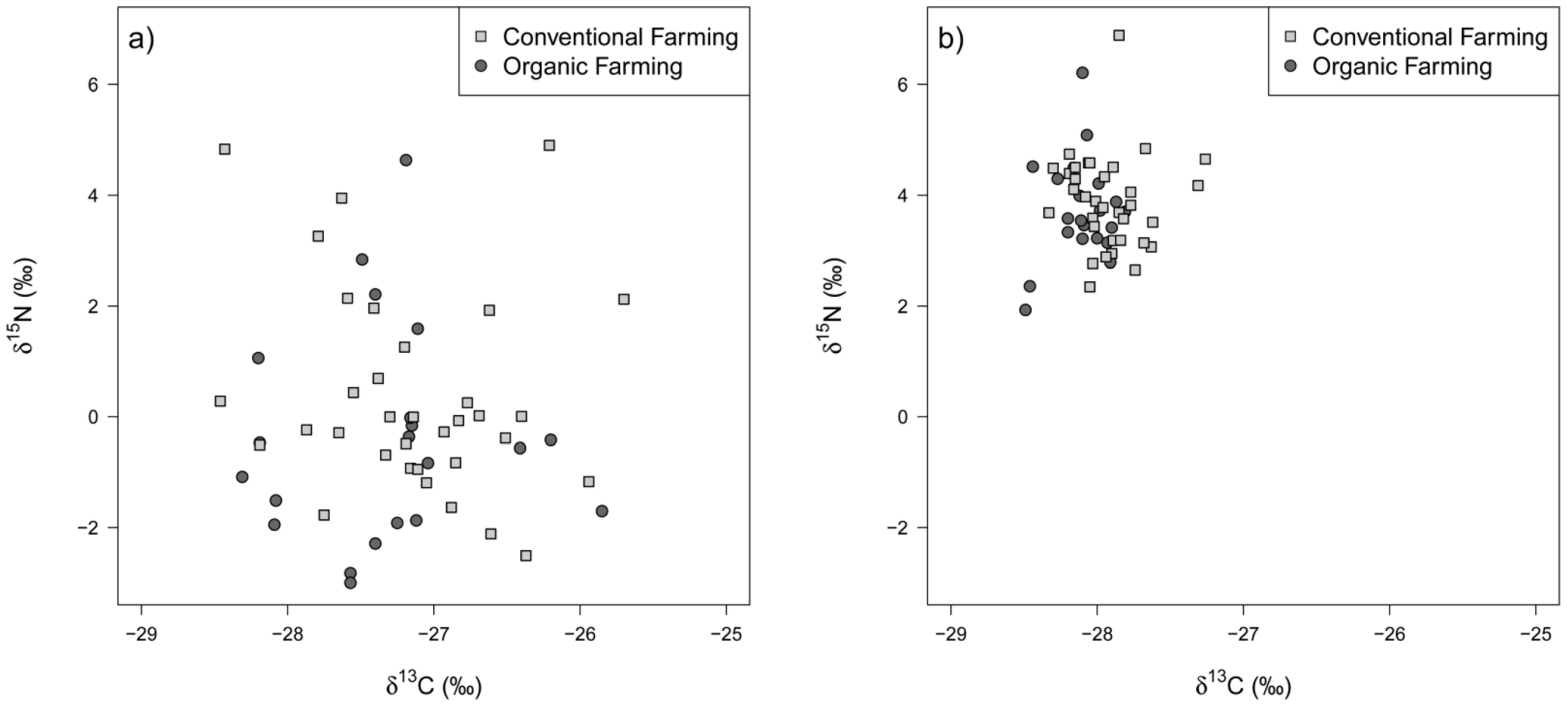
δ^15^N and δ^13^C composition of a) hay and b) soil samples of organic and conventional grasslands.

**Figure 2:**
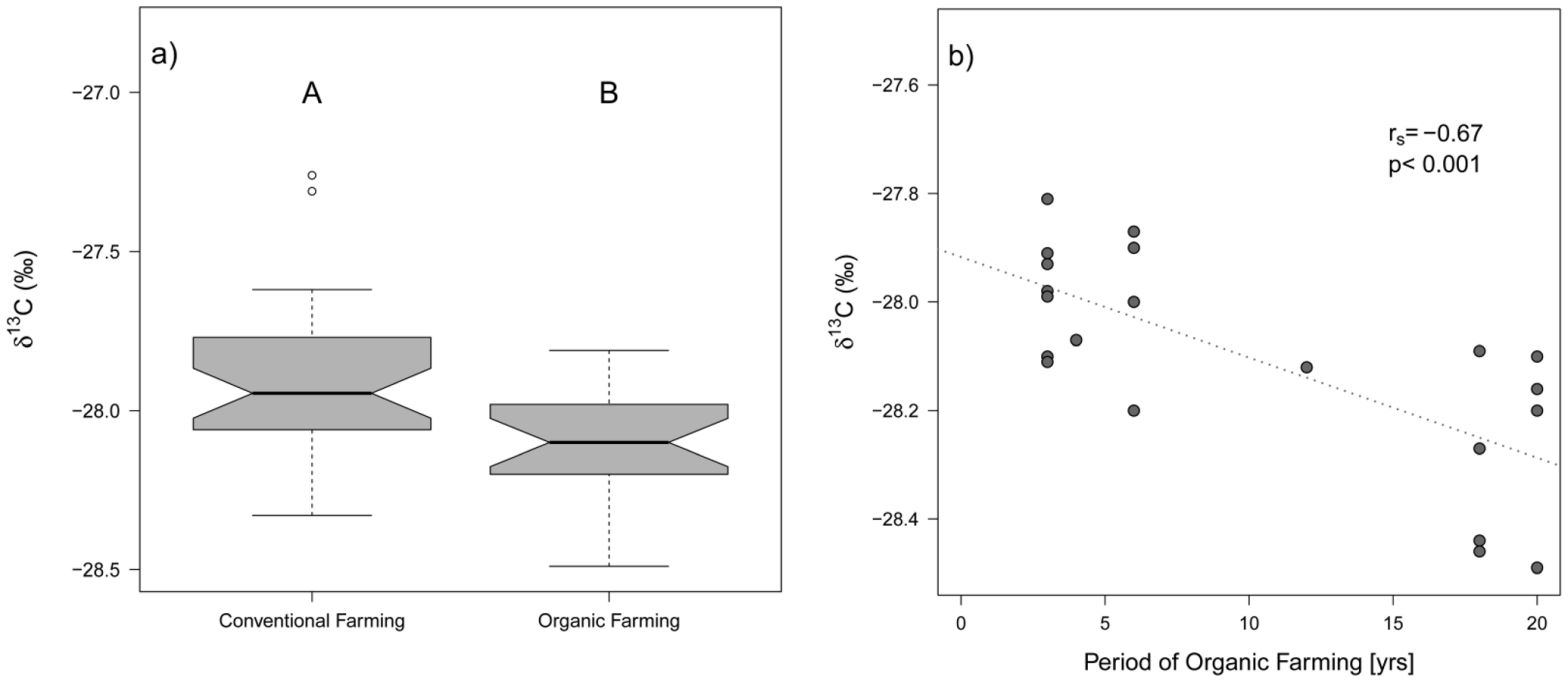
δ^13^C abundances in soil of a) organic vs. conventional grasslands and b) organic grasslands in relation to the time since certification (*r*_s_ = −0.70; *p*<0.001). Letters indicate significant group differences according to ANOVA analyses (for details see Table 1 & 2).

Grasslands of both management types overlap widely in ^13^C and ^15^N isotopic abundances in soil and hay (Figure 1). Nevertheless, organic grasslands were characterized by significantly lower δ^13^C abundances in soil (Figure 2a). Meanwhile, δ^13^C in hay, δ^15^N in hay and soil and Δδ^15^N did not differ between organic and conventional grasslands but only among farms, study regions and/or grassland types (Table 1). In none of the models the soil type was a significant predictor of ^13^C and ^15^N isotopic signals. Although this does not mean that isotopic signals are independent of further soil characteristics, it, nevertheless, underlines the comparability of the selected plots. Generally, analyses explained only 6 to 34% of the variance (Table 1).

**Table 1:** Summary of multiple ANOVA models of isotopic abundances (no interaction with management was significant) (*n* = 55). “Grassland type”  =  pasture, meadow or mown pasture.^a^

	Adj. *R* ^2^	Effect of organic management	Farm	Region	Grassland type	Soil type
**δ13C soil**	0.13**	negative**	ns	ns	ns	ns
**δ13C hay**	0.34***	ns	*	***	ns	ns
**δ15N soil**	0.06*	ns	ns	*	ns	ns
**δ15N hay**	0.24*	ns	*	ns	*	ns
**Δδ15N**	0.20*	ns	**	ns	*	ns

a Significance levels: ***  = *p*<0.001; **  = 0.001<*p*<0.01; *  = 0.01<*p*<0.05.

When related to the time since certification, ^13^C in hay and soil showed significant negative relationships, while the explained variance of the models increased considerably compared to previous models (Table 2). This was most significant for δ^13^C in soil (Figure 2b). Neither δ^15^N in soil or hay nor Δδ^15^N showed significant relationships with the time since certification (Table 2).

**Table 2:** Summary of ANCOVA models of isotopic abundances testing for relationships with the time since certification (only organic plots, *n* = 21).

	Adj. *R* ^2^	Effect of time since certification	Farm	Region	Grassland type	Soil type
**δ13C soil**	0.49***	negative***	ns	ns	ns	ns
**δ13C hay**	0.73**	negative**	*	ns	ns	ns
**δ15N soil**	ns	ns	ns	ns	ns	ns
**δ15N hay**	ns	ns	ns	ns	ns	ns
**Δδ15N**	ns	ns	ns	ns	ns	ns

a Significance levels: ***  = *p*<0.001; **  = 0.001<*p*<0.01; *  = 0.01<*p*<0.05.

“Grassland type”  =  pasture, meadow or mown pasture.^a^

Due to significant effects of study region on some of the isotopic abundances (Table 3), quadratic discriminant analysis (QDA) was carried out using data which was centered (standardized) according to the regional mean. Using QDA, 60% of the samples could be correctly classified as organic or conventional using δ^13^C and δ^15^N isotopic information of soils, while 70% could be correctly classified using the isotopic information of the hay, assumingly due to higher variation in isotopic values of hay compared to soil samples. Combining both, increased correct classifications only slightly up to 73%. In all cases analyses mismatched organic samples to a higher degree than conventional samples and classified significantly more organic samples wrongly as conventional ones (Table 3).

**Table 3:** Results of quadratic discriminant analysis (QDA) of management types (organic vs. conventional) deduced from regionally standardized δ^15^N and δ^13^C isotopic abundances of soil and/or hay samples of grasslands.

Soil samples		Classified			
		organic	conventional	total	correct
** Origin**	organic	7	14	21	33%
	conventional	8	26	34	76%
				**total**	**60%**

Hay samples		Classified			
		organic	conventional	total	correct
** Origin**	organic	9	12	21	43%
	conventional	4	30	34	88%
				**total**	**71%**

Soil & hay samples		Classified			
		organic	conventional	total	correct
** Origin**	organic	10	11	21	48%
	conventional	4	30	34	88%
				**total**	**73%**

## Discussion

Isotopic abundances of ^15^N and ^13^C can give insight in ecosystem functioning and were often shown to be a useful tool to separate between organic and conventional products [1], [6]. In the case of organic management in grasslands, this is only partly true for δ^15^N and δ^ 13^C in soil and hay.

### ^15^N in organic grasslands

Organic grasslands received only organic fertilizers and were characterized by significantly lower fertilization intensity, in line with Klaus et al. [33]. Nevertheless, this difference in fertilization regime did not imprint in the δ^15^N signal of soil and hay samples. While δ^15^N abundances of conventional synthetic fertilizers vary between −2 and 2‰, organic fertilizers such as cattle dung or slurry are strongly enriched in δ^15^N (5 to 35‰) [15]. While experimental studies revealed an effect of organic compared to synthetic fertilization on the δ^15^N isotopic composition of grassland soils and vegetation [23], it was likewise shown that both organic but also mineral fertilizers can lead to increasing δ^15^N vales in soil and vegetation probably due to increased microbial activity and subsequent losses of ^15^N depleted N from the system [17]. Furthermore, conventional management includes both the use of organic and mineral fertilizers and farmers may considerably change respective proportions among years [26]. In our study, the naturally diverse abiotic conditions such as strong variation in soil properties and land-use history among plots might have additionally impeded clear patterns of δ^15^N in organic and conventional grasslands. This is supported by findings from Wrage et al. [34] giving poor to missing associations among N balances and δ^15^N in soil and vegetation due to spatially heterogeneous conditions and short-term changes in stocking densities in pastures. Moreover, as only a certain proportion of the N stored in biomass and soil originates from fertilization, biologically fixed N (e.g. through legumes) have additionally diluted isotopic signals of fertilizer applications [35].

Missing differences in Δδ^15^N between organic and conventional plots suggest that the studied grasslands do not substantially differ in nitrogen cycling [24]. Instead, the respective farm (or land owner) was responsible for most of the variation explained in Δδ^15^N indicating that nitrogen cycling is strongly driven by individual decisions and practice at the farm level. Because plant species richness was shown decease Δδ^15^N in plant and soil assumedly via nitrogen partitioning [35], missing or poor differences in plant diversity between organic and conventional grasslands [e.g. 33,36,37] could be another reason why Δδ^15^N did not respond to organic grassland management. Contrary, in our study organic grasslands had higher mean plant species richness compared to conventional ones (t-test; *p*<0.01; data not shown), apparently not leading to significant differences in Δδ^15^N.

Haas et al. [21] suggest that lower fertilization intensity under organic management is associated with fewer losses of N to neighbouring habitats or to the ground water. However in our case, even after up to 20 years of organic farming ^15^N isotopic abundances revealed no significant indication for fewer losses of N from the studied organic grasslands.

### ^13^C in organic grasslands

We found significant differences in δ^13^C abundances between organic and conventional soils and trends in decreasing δ^13^C abundances with time since certification in both organic soil and hay samples. Missing statistical significances of soil types in the analyses suggested that this is not only caused by differences in water availability among study plots. However, clear differences in δ^13^C in hay might only get apparent in dry years, because δ^13^C isotopic patterns can differ considerably among seasons [38]. Thus, δ^13^C abundances in soils are probably a more reliable indicator for water supply for plants, because they integrate δ^13^C signals over a longer period of time, while δ^13^C in hay strongly depends on the photosynthetic activity and stomatal conductance influenced by current weather conditions [2], [38]. However, it is questionable whether differences in δ^13^C in hay can get more pronounced than in the studied year as prior to sampling the weather was particularly dry.

It was shown that vegetation δ^13^C decrease with plant diversity in grassland experiments due to facilitation and/or complementarity among plant species [39], [40]. Assumedly, we also detected such a pattern of a positive feedback of higher plant diversity under organic management causing less water stress in organic grasslands. However, as mentioned above, organic and conventional grasslands do not always differ in plant diversity and more research is needed to give full evidence for reduced water stress under higher plant diversity in established permanent grasslands. Furthermore, changes in δ^13^C with time since certification cannot be traced back to changes in plant diversity, because diversity was stable over time (data not shown).

There are several other mechanisms, which could have also occurred, but cannot be easily separated from the whole complex of factors influencing isotopic abundances. Georgi et al. [6] found organic biomass (vegetables) to be depleted in δ^13^C. They suggest higher soil respiration rates in organic soils due to higher microbial activity might cause a lowering of the δ^13^C value of the soil CO_2_ pool available to plants. As soil density fractions can have different ^13^C signals [41], differences in the distribution of soil carbon among density fractions could also have also led to isotopic patterns.

Other reasons for lower δ^13^C values in organic grasslands could be direct or indirect effects of (former) fertilization. As organic farms use less often corn (a C4 plant) to feed their livestock, conventional slurry is thus enriched in ^13^C and may have led to higher δ^13^C values in soil organic matter in conventional grasslands [42]. As the “conventional” organic matter deceases after time since certification, this effect is likely to have caused decreasing δ^13^C in soil with time since certification. Similarly, it was shown that high N availability can lead to higher δ^13^C in plant biomass mostly due to structural changes in plant tissue further tightening drought conditions [43]. However, differences in δ^13^C values of vegetables observed by Georgi et al. [6] were independent of optimal or reduced N supply. Finally, we can’t rule out that further factors such as the local rainfall distribution also influenced observed δ^13^C abundances.

### Classification of organic and conventional samples

Although Rapisarda et al. [6] showed that approximately 90% of fruit samples could be correctly categorized as organic or conventional products using isotopic abundances, classification of grassland samples revealed rather weak results. Only one third of organic and conventional samples were correctly classified and a high proportion especially of organic samples were mismatched. This can be largely explained by missing differences in especially δ^15^N abundances. These were significantly higher in organic fruits used in the study by Rapisarda et al. [6]. Thus, we have to conclude that separating organic and conventional soil and hay samples is barely possible using only δ^15^N and δ^13^C abundances. This is especially true, if we would also include uncertified but unfertilized grasslands, which occur frequently [33], [44]. However, including further stable isotopes of S, O and H or other indicative substances might offer additional possibilities to improve the classification of organic vs. conventional samples from permanent grasslands [11], [12].
